# Paternal obesity: how bad is it for sperm quality and progeny health?

**DOI:** 10.1186/s12610-017-0064-9

**Published:** 2017-10-26

**Authors:** Georges Raad, Mira Hazzouri, Silvia Bottini, Michele Trabucchi, Joseph Azoury, Valérie Grandjean

**Affiliations:** 1 Azoury-IVF clinic, Mount Lebanon Hospital, 5th floor, Camille Chamoun bvd, Beirut, Lebanon; 2 Faculty of Sciences 2, Lebanese University, Fanar, Lebanon; 3 Université Cote d’Azur, Inserm, C3M (team 10), Nice, France

**Keywords:** Obesity, Fertility, Inheritance, Epigenetics, Small RNAs, obésité, fertilité, hérédité, épigénétique, petits ARN, sperm

## Abstract

There is substantial evidence that paternal obesity is associated not only with an increased incidence of infertility, but also with an increased risk of metabolic disturbance in adult offspring. Apparently, several mechanisms may contribute to the sperm quality alterations associated with paternal obesity, such as physiological/hormonal alterations, oxidative stress, and epigenetic alterations. Along these lines, modifications of hormonal profiles namely reduced androgen levels and elevated estrogen levels, were found associated with lower sperm concentration and seminal volume. Additionally, oxidative stress in testis may induce an increase of the percentage of sperm with DNA fragmentation. The latter, relate to other peculiarities such as alteration of the embryonic development, increased risk of miscarriage, and development of chronic morbidity in the offspring, including childhood cancers. Undoubtedly, epigenetic alterations (*ie*, DNA methylation, chromatin modifications, and small RNA deregulation) of sperm related to paternal obesity and their consequences on the progeny are poorly understood determinants of paternal obesity-induced transmission. In this review, we summarize and discuss the data available in the literature regarding the biological, physiological, and molecular consequences of paternal obesity on male fertility potential and ultimately progeny health.

## Résumé

De plus en plus de données tendent à montrer que l’obésité paternelle a non seulement des effets néfastes sur la santé métabolique et reproductive de l’individu mais également sur celle de sa descendance. Les mécanismes mis en jeu dans ce processus incluraient des altérations physiologiques et hormonales des fonctions reproductives de l’homme obèse ainsi que des altérations épigénétiques au niveau du génome spermatique. Les modifications hormonales associées à l’obésité et qui se caractérisent principalement par une réduction du taux d’androgènes et une augmentation du niveau d’estrogène induiraient une altération des paramètres spermatiques, une diminution de la concentration ou de la numération totale en spermatozoïde et du volume séminal. Le stress oxydatif dans le testicule induirait une augmentation de la fragmentation de l’ADN spermatique et pourrait rendre compte de l’augmentation des risques de fausses-couches, des problèmes de développement embryonnaire ainsi que de l’augmentation des risques de mortalité chez la descendance, problèmes fréquemment rencontrés lorsque le père est. obèse. Les modifications épigénétiques (altérations des profils de méthylation de l’ADN, de la structure de la chromatine ou/et des profils d’expression des ARN spermatiques) induites par l’obésité sont, quant à elles, loin d’être comprises, même si elles sont, surement, les vecteurs clés de la transmission épigénétique paternelle des maladies métaboliques. L’objet de cette revue est. de résumer puis de discuter les différentes études expérimentales et épidémiologiques publiés à ce jour sur les conséquences physiologiques et moléculaire de l’obésité paternelle sur la santé de l’individu et sur celle de sa descendance.

## Background

Spermatogenesis is a tightly regulated process allowing the production of highly specialized cells, the spermatozoa. In order to give rise to viable progeny, spermatozoa requires two pivotal properties: while the first one is to fertilize oocytes to produce a diploid cell, the zygote, the second one is to bring to the zygote half of its genetic and epigenetic components. Consequently, we can presume that factors that modify the entire physiology of an individual might have negative impact on the quality of its sperm, for example the case of obesity.

As we know body weight homeostasis (from the Greek ὅμοιος, hómoios - meaning ―same and στάσις (ἡ), stásis - meaning ―standing) is crucial for protecting against weight fluctuations. Hence, this equilibrium is adjusted by the metabolic energy network and the control of food intake. However, an imbalance between the energy intake and energy expenditure leads to body weight fluctuations (e.g., gain or loss). The most common weight fluctuations are overweight and obesity, defined as an excessive accumulation of adipose tissue that is either generalized or localized within the body [1, 2]. Two modalities can be used to assess corporal obesity: (i) the measurement of the waist circumference (measurement at the top of iliac crest according to National Health and Nutrition Examination Survey (NHANES) or (ii) the meansurement of the midpoint between the last palpable rib and top iliac crest according to the World Health Association (WHO)) and the calculation of the body mass index (weight/height ratio, BMI (body mass index) = weight (Kg) / height (m^2^) of an individual, allowing to approximately determine the percentage of fat in the body) [3]. Based on the latter ratio, individuals can be grouped into the following six categories: underweight, normal weight, overweight, obese class I, class II and class III (Table 1). Recent epidemiological studies have reported a striking increase prevalence of obesity worldwide. It is noteworthy to mention that these values reaches alarming percentages, for example 34.9%, 36% and 28.2% in USA, Saudi Arabia, and Lebanon respectively [4–7]. Strikingly, we pointed a high prevalence of 13% in young men of reproductive age suffering from overweight and obesity [8, 9]. Besides other known pathologies associated to obesity, such as hypertension, kidney disease, and type 2 diabetes, male infertility linked to obesity has sparkled the interest of the research community [10]. While several studies indicated that obesity could negatively impact sperm quality through physiological alterations (such as hormonal profiles modification (*ie* reduced androgen levels accompanied by elevated estrogen levels) [11, 12], and molecular alterations (such as epigenetic modifications which may have health cues in the offspring) [13–16], other studies did not support completely this association [17, 18]. Accordingly, we elected to review and discuss the impact of paternal obesity on the male reproductive health as well as on the metabolic health of the progeny.

**Table 1:** Table showing the classification of the overweight and obesity [147].

Categories	Body mass index ( Kg/m^2^)
Underweight	< 18.5
Normal weight	18.5-24.9
Overweight	25.0-29.9
Class I obesity	30.0-34.9
Class II obesity	35.0-39.9
Class III, extreme obesity	≥ 40

To write this review, we systematically collected papers published until September 2017, by searching Pubmed (URL:http://www.ncbi.nlm.nih.gov/pubmed/) and using keywords (such as epigenetic inheritance, sperm, obesity) related to the study background. This search was limited to English-languages publications.

## Paternal obesity and male reproductive health

### Effect of obesity on sexual function and seminal plasma composition

Sedentary lifestyle and increase of calories intake by obese men could impair their reproductive health in different ways. On one hand, obese men are at higher risk of facing erectile dysfunction and reduced libido [19–21]. Interestingly, these functions were demonstrated to be restored after weight loss [22]. On the other hand, the sex accessory glands and the seminal plasma components could also be affected by male obesity [23]. The seminal plasma is an alkaline gelatinous fluid (pH ~7.2), made up of seminal vesicle secretions (~60% of the total semen) and prostate gland secretions (~20% of the total semen volume). The secretions of the different sex glands contribute to the complex content of the seminal plasma [24]. This biological fluid could regulate sperm physiology by several ways such as: providing the energy source for spermatozoa [25], inducing biochemical modification of sperm during capacitation [26], and regulating acrosome reaction 4 [27]. Particularly, it was shown in mice that the excision of the seminal vesicle reduced the percentage of spermatozoa with progressive motility, accordingly this will drastically affect in the female uterine cavity, thus decreasing the pregnancy rate in this model [28].This study highlighted the importance of the seminal fluid as a transport and survival medium for spermatozoa. Furthermore, the seminal fluid can also modulate the female reproductive tract, and this finding was illustrated by several studies performed in mice, pigs, and humans [29]. For instance, it was shown that the excision of the seminal vesicle in a mouse model induced in part the up-regulation of some embryotrophic factor genes and the down-regulation of an apoptosis- inducing factor in the oviduct [30]. In addition, the absence of the seminal vesicle secretions impaired the blastocyst development and severely affected the resulting progeny that exhibited obesity, intolerance to glucose, and hypertension [30]. These results strongly indicated that the seminal fluid components regulate gene expression in the oviductwhich influences embryo development and progeny health. In addition, several studies in mammals highlighted the role of seminal plasma in regulating fetus growth trajectory [31–33]. Particularly, in a mouse model of diet-induced obesity, Binder and colleagues showed that seminal vesicle proteins and metabolites are affected by obesity [34]. Interestingly, clinical data reported that obese men are at higher risk of having a reduced semen volume and an alterated seminal plasma biochemistry compared to non-obese men [35]. Notably, the level of insulin, leptin, fructose, and interleukin 8 were found to be high in the semen of obese men. Conversely, adiponectin, progranulin, and alphaglucosidase levels were lower in obese men compared to lean men [36–39]. These alterations may affect the sperm biology by several ways. For instance, fructose is secreted by the seminal vesicle, and it is the major energy substrate in the semen. The level of seminal fructose was shown to be altered by several etiologies of male infertility [40, 41]. Plus, a positive correlation was found between seminal fructose and sperm DNA fragmentation [42]. Thus, altered seminal fructose level in obese men probably reflects an altered seminal vesicle function [43]. Furthermore, the receptors of leptin and insulin were shown to be present on the sperm plasma membrane [44]. Probably, the alterations of the level of these two hormones in the seminal plasma of obese men may alter the endocrine signaling inside the spermatozoa. In parallel, it was demonstrated that the seminal plasma initiates changes in the cytokine profile of the uterine cavity and thus influence early embryo development and implantation [45]. For this reason, high levels of interleukin-8 in the semen of obese men may alter the inflammatory response in the uterine cavity and may impair implantation and progeny health. Overall, these data opens new avenue to investigate the drawbacks of an altered seminal plasma composition in obese men on spermatozoa, the uterine environment, and embryo development.

### Effect of paternal obesity on testis

**Fig. 1:**
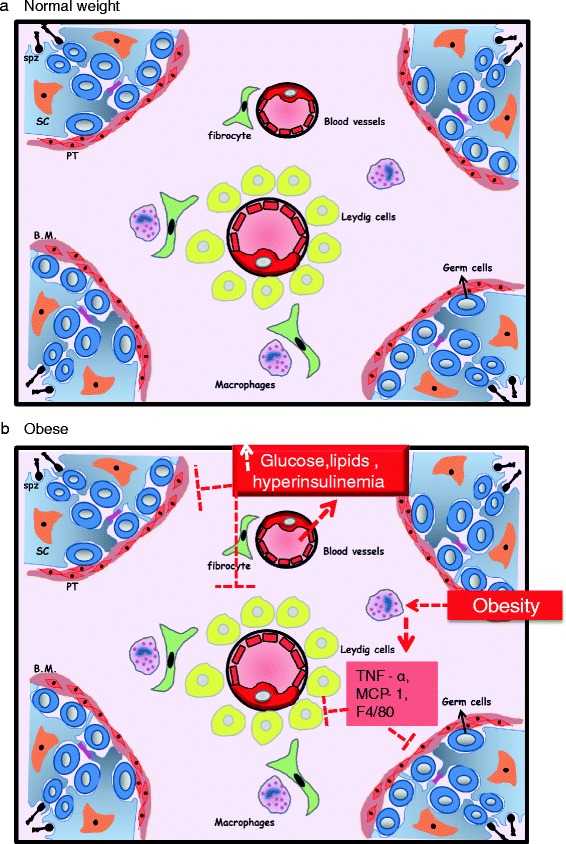
Illustration of testicular interstitial tissue in normal weight men (**a**) and obese men (**b**). Spz = Spermatozoa; sc = Sertoli cells; PT = peritubular cells; B.M. = basement membrane. TNF-alpha: Tumor necrosis factor alpha; MCP-1 = monocyte chemoattractant protein-1; F4/80: a defining marker of murine macrophage populations

Basically, the human testis is divided by a group of septa into 250 to 300 lobules. Each lobule is composed of interstitial tissue and 1–3 seminiferous tubules. While the interstitial and the tubular compartments are histologically distinguishable from each other, they are physiologically connected. All the processes involved in the production of male gametes (sperm cells) take place in the tubular compartment. However, the interstitial tissue is composed of Leydig cells (200 x10^6^Leydig cells per testis), immune cells, blood vessels, fibroblasts, and connective tissue, and its main function is the production of male sex hormones. The integrity of both compartments is crucial for male gametes differentiation (Fig. 1) [46].

### Effect of obesity on Leydig cell physiology and androgen homeostasis

Firstly described in 1850 by Franz Leydig, Leydig cells secrete the most important male steroid hormone called testosterone via steroidogenesis. These cells can easily access to the blood vessels, which permits the uptake of the luteinizing hormone (LH) and cholesterol from the circulation in order to produce testosterone. The produced male sexual hormone diffuses into the interstitial and tubular compartment to regulate spermatogenesis. In addition, when testosterone is released into the bloodstream it plays various roles in brain masculinization and sexual behavior, modulation of the larynx growth, stimulation of erythropoietin synthesis in the kidney, maturation of male sexual organs, hair growth, regulation of the bones muscle mass, and protein synthesis in the liver [47].

**Fig. 2:**
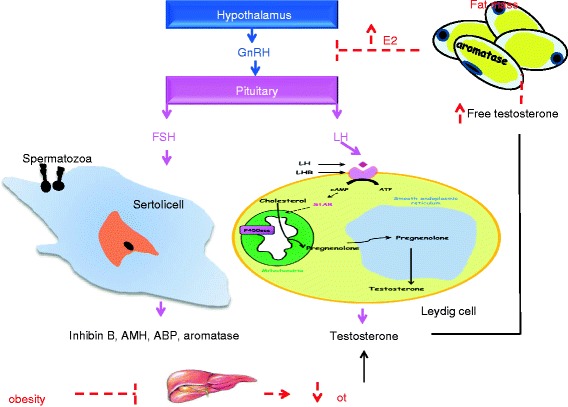
Schematic representation of the hypothalamic pituitary testicular axis and hormone testicular production upon obesity. Solid lines represent the hormonal regulation in normal weight men; dashed lines represent the inhibitory effects of obesity. AMH: anti-müllerien hormone; ABP: androgen binding protein; E2: oestrogen; FSH: follicle stimulating hormone; GnRH: gonadotropin releasing hormone; LH: luteinizing hormone; LHR: luteinizing hormone receptor; SHBG: sex hormone binding globulin; StAR: steroidogenic acute regulatory protein

Furthermore, testosterone indirectly can regulate the action of the hypothalamus-pituitary-testis axis. In fact, male hypothalamus releases the gonadotropin-releasing hormone (GnRH) that will bind to its receptor on the pituitary gonadotroph cells stimulating the expression and the release of LH and follicle-stimulating hormone (FSH) [46]. LH stimulates the testosterone production by the Leydig cells by binding to its receptor (LHR) on the Leydig plasma membrane [48]. The circulating testosterone is mainly bound to albumin or to sex hormone binding globulin (SHBG) which is originally synthesized in the liver. SHBG was demonstrated to reduce the clearance rate of androgens and regulates their access to target tissues [49]. In addition, the free circulating testosterone can be transformed in the adipose tissue into estrogen (E2) by an enzyme called aromatase (Fig. 2) [50]. Notably, in obese men, excess fat mass and hyperinsulinemia may alter SHBG production in the liver. Therefore, this will increase the amount of free testosterone available for conversion into E2 in the accumulated fat mass, which in turn will reduce GnRH secretion in the hypothalamus [51–53]. The above-mentioned pathophysiological changes could explain the increase in E2 concentration and the decrease in testosterone and gonadotropins in obese men [12, 53]. It is known that, glucose and lipid homeostasis are essential for normal Leydig cells function. Thus, any alterations in glucose levels or consumption of high amounts of unhealthy fats can inhibit testosterone production and induce Leydig cells apoptosis [54–56].

### Effect of obesity on Sertoli cells functions

Sertoli cells play a crucial role in germ cells development as well as in the regulation of spermatogenesis. These cells receive hormonal messages (FSH and testosterone) and local signals (autocrine and paracrine) to secrete molecules modulating their own function as well as that of germ cells and Leydig cells [47].

It was shown that the hormones involved in the regulation of spermatogenesis (FSH) and secreted by the Sertoli cells (inhibin B, AMH) were lower in obese men when compared to non-obese individuals [53, 57, 58]. Moreover, obesity may alter Sertoli cell metabolism which will affect germ cells. On the one hand, insulin stimulates several activities in Sertoli cells, mainly carbohydrate metabolism [59]. Lactate is produced by Sertoli cells under the control of insulin. It is the main energy source for germ cells, in addition ofhaving an anti-apoptotic effect [60, 61]. Rato et al. showed that feeding rats with a high-energy diet increased the expression of glucose transporters and glycolytic enzymes in Sertoli cells. Altogether, these changes increased the level of lactate leading to oxidative stress in these cells [56]. In addition, testicular biopsies from diabetic men showed vacuolization of Sertoli cells. These observations may alter testicular glucose homeostasis (Fig. 1b) [62]. On the other hand, lipid metabolism also plays an important role in the testicular tissue function. Sertoli cells can uptake fats through various modes, either by passive diffusion through the plasma membrane or by protein facilitated transport, or from phagocytosed cells. Sertoli cells may utilize the internalized lipids to produce energy (ATP) or to generate polyunsaturated fatty acids (PUFAs). The PUFAs are essential for germ cells plasma membrane fluidity and flexibility and thus ultimately for fertilization [63]. The excessive consumption of saturated fats present in unhealthy diets may lead to their accumulation in testicular cells. This will affect the fatty acid composition of plasma membrane and the process of spermatogenesis [56, 64].

It is important to note that, the blood testis barrier (BTB) can modulate to a certain extent glucose and lipid uptake depending on their availability in blood [62]. However, histological analysis of testis from mice on high fat diet demonstrated that obesity can compromise the BTB integrity [65]. In the same perspective, obesity may bypass the BTB adaptive mechanisms to changing environmental conditions and thus targeting its main functions in selective transport and permeability [62].

### Effect of obesity on spermatogonia

The spermatogonia are the final germline stem cells rising from primordial germ cells (PGC). They are characterized by a self-renewal capacity, and their commitment to spermatogenesis. Male obesity could also impair the survival and the differentiation of these cells [66]. Of particular interest, Interleukin 6 (IL-6) is a cytokine secreted by adipose tissue and macrophages. At elevated concentrations, it induces an inflammatory response. A recent study has shown that a high level of IL-6 is detected in the serum and testis of obese mice. We could speculate that this rise may decrease the level of a zinc finger protein (Zfp637) in spermatogonia. The downregulation of Zfp637 reduces spermatogonial differentiation [67]. Furthermore, obesity may induce testicular hyperthermia. It was found that specific spermatogonia (Adark) are more vulnerable to heat stress due to their high mitotic activity [68, 69]. Altogether, these data highlight the negative impact of obesity on spermatogonial survival and differentiation.

## Impact of paternal obesity on sperm parameters and embryo development

### Paternal obesity negatively affects the sperm parameters

Many systematic reviews were performed in humans to evaluate the effects of male obesity on sperm parameters, DNA fragmentation, mitochondrial membrane potential, and fertility outcomes [11, 18, 70–72]. Although some discrepancies were noted [11, 18], overall these studies indicated that overweight and obesity in male are associated with high incidence of oligozoospermia and azoospermia [73–75], reduction in the percentage of normal sperm morphology [11, 18], increased percentage of sperm with DNA fragmentation and abnormal mitochondrial membrane potential [11, 75, 76]*.*

Some of these complications were found linked to testicular oxidative stress [77]. In fact, spermatogenesis is associated with a high rate of oxygen consumption and a resulting important ROS production by mitochondria causing oxidative stress [78]. The hyperglycaemia and hyperlipidemia would exacerbate this outcome. Indeed, intracellular accumulation of lipids increases β-oxidation rate of fatty acids. When overloaded, stressed testicular mitochondria reduce ATP production, and induce ROS overproduction [56]. More so, high-energy diets would reduce the testicular antioxidant system by reducing the expression of ROS-detoxifying enzymes in the testis such as proliferators-activated receptor γ coactivator 1α(PGC-1α) and sirtuin 3 (SIRT3) [79]. Not surprisingly, studies in humans and rodents have demonstrated that obesity is associated with increased sperm ROS production [80, 81]. High ROS can produce important destructive effects in tissues by causing alterations in membranes causing an irreversible cellular damage. On the other hand, elevated ROS levels can induce DNA damage in spermatids [81] and in mature sperm [82–86]. Altogether, these findings reveal that obesity induces an oxidative stress in the testis and thus impairs sperm quality [56].

### Paternal obesity and embryo development

It was demonstrated that obesity in rodents has a negative impact on pre-implantation embryo development, in particular we pointed a higher percentage of one –cell embryo block, delayed cell cycle progression, decreased blastocyst number, and altered carbohydrates metabolism [87, 88]. Regarding human in vitro embryo development, very few reports were published. Whereas two studies didn‘t find any significant difference between obese and non-obese men regarding embryologic parameters, Bakos et al. spotted a significant decrease in blastulation rate with increasing BMI [84, 89, 90]. Strikingly, a recent meta-analysis included 115,158 participants, showed that paternal obesity may reduce the live birth rate per assisted reproductive technology (ART) cycle, and increases by 10% the risk of facing a non-viable pregnancy [11]. These findings highlighted the negative impact of obesity on the embryonic development.

## Paternal obesity and consequence on the metabolic health progeny

A new area of research in andrology shed light on the long-term consequences of paternal health, at the time of conception, on the health of the offspring. For example, paternal age, smoking, and exposure to toxic chemicals may increase the risk of neuropsychiatric disorders, metabolic alterations, respiratory tract infection, and cancer in the offspring [91–97]. Similarly, accumulating epidemiological studies in humans suggest that paternal body mass index (BMI) may influence the health of the next generation [32, 98–102]. As reported by Kaati et al., when paternal grandfather experienced a surfeit of food at the age of 8–12 years old, the risk of grandson’s death by T2DM increased by 4 folds. On the other hand, the risk of death by cardiovascular complications was reduced in case of low food availability during father’s young age [100]. However, in research using human clinical samples the amount of material available is most often scarce as well as for ethical reasons, molecular mechanisms involved in this process are still largely unknown. Although human genetics and epigenetics epidemiological studies provided some clues on these mechanisms, however, the high degree of individual genetic/epigenetic variation render the interpretation of the data very complicated. Based on recent significant progress in rodent models [15, 103–113], we can now propose several mechanistic hypotheses. We will describe these molecular mechanisms in the next sections.

### Possible mechanisms of intergenerational and transgenerational inheritance of paternal acquired obesity

Modifications known to be part of the epigenome, namely DNA methylation, chromatin structure, and non coding RNA might be involved in the molecular mechanism of this process [114].

#### DNA methylation and epigenetic inheritance

The chemical modification of DNA by the addition of a methyl group to the 5 position of cytosine was first discovered in 1948 by Rollin Hochkiss [115]. This modification is generally associated with long-term transcriptional repression [116]. Male and female gametes, like all cells, contain a specific DNA methylation landscape. Following fertilization, the majority of these modifications are erased to generate a totipotent zygote. This process is called epigenetic reprogramming [117]. However, some specific sequences escape this reprogramming process, and that is clearly the case of most of imprinted genes that contain differentially methylated regions regulating their parent of origin-specific expression [118]. This also appears to be the case for specific loci that have been marked following environmental changes such as unhealthy diet [34, 105, 107, 119]. In a cohort of 23 overweight/obese and 44 normal weight men human, Soubry et al. showed that sperm from overweight and obese men had significantly lower methylation rate at several imprinted genes [120]. The same authors previously found altered methylation profiles at several differentially imprinted regions in children born to obese parents [16]. Experiment studies support the association between obesity and DNA methylation alterations in gametes and in the somatic tissues of the progenies of the obese father. Along these lines, Ng Sheau-Fang et al. showed that male rats fed a high fat diet (HFD) gave birth to females (F1) intolerant to glucose with abnormal insulin secretion. These metabolic alterations were also associated with the histopathological changes in the pancreatic islets. At the molecular level, the key islet gene Il13ra2 (interleukin 13 receptor subunit alpha 2) was hypomethylated and its mRNA was found up-regulated in the pancreatic islets of the F1 offspring, suggesting a possible role of DNA methylation in the intergenerational inheritance of the paternal acquired phenotype [107]. In parallel, paternal diet-induced obesity in male mice model (C57BL6) was found to be associated with fetal growth restriction which is characterized by reduced fetal and placental weights. The fetal growth restriction is also correlated with an increased risk of developing obesity and diabetes in adulthood [121, 122]. The molecular analysis of the placentas showed that peroxisome proliferator-activated receptor alpha (Pparα) and caspase-12 (Casp12) expression were significantly down-regulated in male placentas from obese fathers when compared to normal fathers, whereas the global DNA methylation was only increased in female placentas [23]. Furthermore, the paternal acquired obesity in mice alters the total body weight as well as glucose homeostasis in female offspring (F1) and to a lesser extent in males. These phenotypes were also transmitted to the second generation (F2) but in a sex-specific manner. At the epigenetic level, the elongated spermatids of the grandfather‘s testes fed a HFD were significantly hypomethylated when detected by immunohistochemistry on testis sections [105]. Finally, in a recent study, HFD was shown to alter DNA methylation signature of spermatozoa which was partially transmitted to the offspring [104].

While these studies showed that an altered DNA methylation signature of spermatozoa from HFD males could be passed through the progenies [104], a recent study indicated that sperm methylome is shaped by genetic and epigenetic variations but not by diet [123].

#### Chromatin structure and epigenetic inheritance

The majority of sperm DNA (negatively charged) is bound to small basic protamines (P1 and P2, positively charged proteins) causing DNA to coil into toroids [124]. The toroids are packaged into a highly condensed chromatin. Moreover, they are attached to the sperm nuclear matrix by their linker region DNA. The vast majority of the DNA is hidden within the toroids, for protection against nuclease digestion [125]. In addition, protamines cysteine residues can form disulfide bridges via the thiol (−SH) groups, thus increasing the stability of the chromatin. At the functional level, this highly compacted structure represses transcription during spermiogenesis and protects the paternal genome during its journey in the female tract. Moreover, paternal protamines are replaced in the first 2–4 h after fertilization by maternal histones, rendering the chromatin more accessible to transcription machinery [126]. A recent meta-analysis showed that abnormal sperm protamination was associated with male infertility and sperm DNA damage. The normal ratio of P1-P2 is approximately 1. This ratio could be affected by several internal or external factors such as thermal stress and cigarette smoking [127–130]. Not the majority of histones are replaced by protamines, but a small percentage from 5 to 10% of the human sperm genome retains paternal histones. In mature sperm of mice and humans, retained histones and their modifications (e.g., H3K4me3, H4K27me3) are not randomly distributed, and they are not replaced by maternal histones after fertilization [131, 132]. Interestingly, the retained nucleosomes were found in the promoters of different developmental genes (such as Hox-, Fox-, Sox-, gata family genes and noncoding RNAs (micro-RNAs and long non-coding RNAs)) [126, 133, 134]. Altogether, these findings highlight the possible role of nucleosome-bound sperm chromatin as a paternally inherited epigenetic regulator of diet induced obesity. Several studies supported this hypothesis. For example Terashima et al. have demonstrated that paternal acquired obesity can modulate histone composition at specific genes implicated in development and cell fate decision [135]. On the other hand, the histone deacetylases (HDAC) class III or Sirtuin (SIRT1–7) proteins are regulated by caloric intake. Of particular interest, SIRT6 is expressed in the mouse elongating spermatids and can play the role of ADP ribosyltransferase and H3 deacetylase (H3K9 and H3K56). Palmer et al. found that the protein level was significantly decreased in spermatids of HFD-fed male mice compared to controls. Consequently, the percentage of spermatids that stained positive to the H3K9ac antibody was higher in the HFD-fed mice compared to controls [81]. In parallel, low-protein diet was associated with a decrease in the H3K27me3 retention in sperm, specifically in Maoa (Monoamine oxidase) and Eftud1 (Elongation Factor Like GTPase 1) promoters [136].

Overall, these findings clearly show that sperm chromatin could be modulated by dietary conditions and could transfer epigenetic information to progeny.

#### Sperm RNA and epigenetic modification

As described above, sperm is a transcriptionally inactive cell which has long been thought to be devoid of RNA. However, several RNA populations including small non-coding RNAs such as microRNA (miRNAs), endogenous small interfering RNAs (endo-siRNAs), Piwi-interacting RNAs (piRNAs), were recently detected in sperm [137]. Since their discovery, several studies demonstrated their role in both early embryogenesis and epigenetic inheritance [138, 139]. For instance, using intra cytoplasmic sperm injection experiments, Lui et al. found that sperm partially deficient in sperm-borne miRNA and endosiRNA could successfully fertilize. However, embryos derived from these sperms displayed different embryonic alterations [139]. On the other hand, sperm-borne microRNA-34c was only detected in spermatozoa and zygotes but not in oocytes. It has been shown to be important for the one-cell embryo DNA synthesis and the first cleavage division [138]. Altogether, these data demonstrated the potential role of small RNA in embryonic growth.

Few years ago, we provided the first evidence that small non-coding RNA molecules (sncRNAs) act as trans-generational vectors of epigenetic information in mice. Indeed, we demonstrated that microinjection of specific microRNA into one cell embryo induced stable epigenetic modifications leading to specific and inherited phenotypes. Thus, cardiac hypertrophy, abnormal adult growth, and fur depigmentation can be induced by the microinjection into a mouse fertilized oocytes of miR-1, miR-124, and miR-221 respectively. Importantly, all these phenotypes can be maintained for at least 2 successive generations [140–142].

To further determine whether sperm RNAs would be critical determinants of the inheritance in an acquired disorder, micro-injection experiments were performed into one-cell embryos with sperm RNA from male mice exposed or not to environmental changes. Thus, sperm RNAs derived from male sperm exposed to postnatal trauma into the zygote, can faithfully reproduce the paternal phenotype in the offspring [143]. Similarly, we demonstrated that mice derived from the microinjection into naive embryos of sperm RNA from HFD-fed mice, even though they had been fed with a control diet, they developed in adulthood diet-induced pathologies such as obesity and signs of diabetes [15]. To identify RNA molecules involved in this epigenetic inheritance, small RNA deep-sequencing analysis of testis RNAs from HFD-fed mice and control was performed and revealed deregulation ofseveral classes of small-RNAs, such as miRNA, piRNA, and fragments of tRNAs. Notably, these results were consistent with previous studies [105]. Importantly, when microinjected into naive zygotes, one of the deregulated miRNA - the microRNA-19b, induced metabolic alterations that were similar to the diet-induced phenotypes [15]. In parallel, two other groups found that tRNA-derived small RNAs (tsRNA) might also contribute to intergenerational inheritance of metabolic disorders [103, 106]. In contrast, synthetic microinjected-tsRNAs did not induce metabolic disorders in the offspring. The authors suggested that only post-transcriptionally modified tsRNAs could mediate thetransgenerational inheritance. This conclusion is supported by the evidence of elevated levels of m^5^C and m^2^G modifications in the tsRNAs of sperms from HFD-fed mice. In addition to the small RNA sperm population, a recent study indicates that long non-coding RNA could also acts as vectors of paternal epigenetic inheritance [108]. Hence, altogether these studies indicate that sperm small RNA is a pivotal paternal epigenetic vector involved in intergenerational inheritance of diet-induced metabolic disorders.

### Potential reversibility of the newly established epigenetic modifications

The advantage of epigenetic alterations over genetic mutations is their potential reversibility [144]. Based on this property, a number of recent experimental studies aimed to demonstrate the reversibility of newly environmental-induced epigenetic modifications. Thus, not only metabolic pathologies but also psychiatric disorders can be epigenetically inherited through the father, they appear to be partially prevented via diet/exercise/environmental intervention in fathers [112] [143, 145, 146]. However, these studies have been performed in rodent experimental models and there is a strong need to expand research on population-based data in order to enhance prevention strategies. To date, only Barrès’ study has raised this issue [13]. In an obese and overweight human cohort, it was firstly demonstrated that environmental stress, such as obesity, might induce epigenetic changes in human spermatozoa. Notably, they, showed that sperm DNA methylation and RNA profiles are different between obese and lean men. More so, they noticed a significant deregulation of piRNA expression. Moreover, they showed in a specific cohort of obese men before and after surgery-induced weight loss a change in sperm methylome. This suggests the reversibility of newly sperm epigenetic diet-induced modifications.

## Conclusions

There is a growing body of evidence supporting that obesity negatively affects sperm quality. Epigenome and small non-coding RNAs alterations in sperm of obese individuals were found to have a significant impact on male fertility potential and the health of the progeny. Owing to the reversibility of such alterations, obese patients are often encouraged to lose weight before being recommended a medical procedure. However, some doubts might be raised about the assumption that a balanced diet could totally or partially reverse epigenetic and small non-coding RNAs alterations.

## Abbreviations

8-OHdG: 8-hydroxydeoxyguanosine
ABP: Androgen binding protein
ADP: Adenosine monophosphate
AMH: Anti-müllerien hormone
ART: Assisted reproductive technology
ATP: Adenosine triphosphate
BMI: Body mass index
BTB: Blood Testis Barrier
Casp12: Caspase-12
E2: Oestrogen
Eftud1: Elongation Factor Like GTPase 1
FSH: Follicle stimulating hormone
GnRH: Gonadotropin releasing hormone
H3K4me3: Tri-methylation (me3) of lysine 4 (K4) on histone H3
H3K56: Lysine 56 of the histone 3
H3K9: Lysine 9 of the histone 3
H3K9ac: Acetylation of lysine 9 (K9) on histone H3
H4K27me3: Tri-methylation (me3) of lysine 27 (K27) on histone H3
HDAC: Histone deacetylases
HFD: High fat diet
Il3ra2: Interleukin 13 receptor submit alpha 2
IL-6: Interleukin 6
LH: luteinizing hormone
m^2^G = N^2^-: methylguanosine
m^5^C = 5-: methylcytosine
Maoa: Monoamine oxidase
MDA: Malondialdehyde
miRNA: microRNA
NHANES: National Health and Nutrition Examination Survey
NO: Nitric oxide
PGC-1α: Proliferators activated receptor γ coactivator 1α
PiIK3r1: Phosphatidylinositol 3 kinase regulatory subunit
PiK3ca: Phosphatidylinositol 3 kinase catalytic subunit
piRNAs: Piwi-interacting RNAs
Ppar: Peroxisome proliferator-activated receptor alpham
PUFAs: Polyunsaturated fatty acids
RNA: Messenger ribonucleic acid
ROS: Reactive oxygen species
SGA: Small for gestational age
SHBG: Sex hormone binding globulin
SHBG: Sex hormone binding globulin
siRNAs: Small interfering RNAs
SIRT: Sirtuin protein
sncRNAs: Small non-coding RNA
T2DM: type 2 diabetes mellitus
tRNAs: Transfer ribonucleic acid
tsRNA: tRNA-derived small RNAs
WHO: World Health Association
Zfp: Zinc finger protein
